# The Myotube Analyzer: how to assess myogenic features in muscle stem cells

**DOI:** 10.1186/s13395-022-00297-6

**Published:** 2022-06-10

**Authors:** Simon Noë, Marlies Corvelyn, Sarah Willems, Domiziana Costamagna, Jean-Marie Aerts, Anja Van Campenhout, Kaat Desloovere

**Affiliations:** 1 Research Group for Neurorehabilitation (eNRGy), Department of Rehabilitation Sciences, KU Leuven, Leuven, Belgium; 2 Translational Cardiomyology, Stem Cell and Developmental Biology Unit, Department of Development and Regeneration, KU Leuven, Leuven, Belgium; 3 M3-BIORES, Division Animal and Human Health Engineering, Department of Biosystems, KU Leuven, Leuven, Belgium; 4 Department of Orthopedic Surgery, University Hospitals Leuven, Leuven, Belgium; 5 Department of Development and Regeneration, KU Leuven, Leuven, Belgium

**Keywords:** Cerebral palsy, Satellite cell cultures, Fusion index, Image analysis

## Abstract

**Background:**

The analysis of in vitro cultures of human adult muscle stem cells obtained from biopsies delineates the potential of skeletal muscles and may help to understand altered muscle morphology in patients. In these analyses, the fusion index is a commonly used quantitative metric to assess the myogenic potency of the muscle stem cells. Since the fusion index only partly describes myogenic potency, we developed the Myotube Analyzer tool, which combines the definition of the fusion index with extra features of myonuclei and myotubes obtained from satellite cell cultures.

**Results:**

The software contains image adjustment and mask editing functions for preprocessing and semi-automatic segmentation, while other functions can be used to determine the features of nuclei and myotubes. The fusion index and a set of five novel parameters were tested for reliability and validity in a comparison between satellite cell cultures from children with cerebral palsy and typically developing children. These novel parameters quantified extra nucleus and myotube properties and can be used to describe nucleus clustering and myotube shape. Two analyzers who were trained in cell culture defined all parameters using the Myotube Analyzer app. Out of the six parameters, five had good reliability reflected by good intra-class correlation coefficients (> 0.75). Children with cerebral palsy were significantly different from the typically developing children (*p* < 0.05) for five parameters, and for three of the six parameters, these differences exceeded the minimal detectable differences.

**Conclusions:**

The Myotube Analyzer can be used for the analysis of fixed differentiated myoblast cultures with nuclear and MyHC staining. The app can calculate the fusion index, an already existing parameter, but also provides multiple new parameters to comprehensively describe myogenic potential in its output. The raw data used to determine these parameters are also available in the output. The parameters calculated by the tool can be used to detect differences between cultures from children with cerebral palsy and typically developing children. Since the program is open source, users can customize it to fit their own analysis requirements.

**Supplementary Information:**

The online version contains supplementary material available at 10.1186/s13395-022-00297-6.

## Background

Patients with neurological disorders, such as cerebral palsy (CP), are characterized by altered muscle morphology. CP is a neuromuscular disorder, characterized primarily by a brain lesion in the immature brain and secondarily by musculoskeletal problems [1]. Literature has described multiple morphological differences at the level of the muscle comparing those from typically developing (TD) subjects and patients with CP [2, 3]. For example, smaller fiber sizes, accumulated extracellular matrix deposition, and lower numbers of satellite cells have been reported for patients with CP [3–5].

A well-known approach to better understand the origin of altered skeletal muscle morphology in patients is to study the in vitro culturing of adult stem cells obtained from muscle (micro) biopsies [6, 7]. While differences between cultures obtained from muscles of patients and healthy controls can be quantitatively assessed via biochemical methods that require a large number of cells, qualitative methods used for smaller numbers of cells do not allow for an adequate quantification of the differences. One possible compromise to assess myogenic potency through immunofluorescence analysis involves the calculation of the fusion index obtained from differentiated satellite cells, the main pool of myoblasts available in the adult muscle [6–9].

The fusion index is commonly used in muscle cell culture assays to determine the amount of myoblast fusion [6–9]. To this end, nuclei are visualized using DNA binding compounds like Hoechst, and myotubes are stained using fluorescent labelled antibodies for structural muscle protein, mainly myosin heavy chain (MyHC) among others. The fusion index is calculated as the number of nuclei inside MyHC-positive myotubes divided by the total number of nuclei present in a field of view. A myotube is therefore defined as a syncytium with an elongated tubular shape, recognizable as an area stained with MyHC antibodies and characterized by the presence of at least two nuclei [6, 10]. This calculation requires both a method to count nuclei and a method to distinguish which nuclei are inside MyHC-positive myotubes and which are not. While the counting of all nuclei in an image is can be performed using (semi-) automatic methods through software applications, such as for example FIJI [11], counting only nuclei inside myotubes is currently done manually, requiring a lot of time from an expert researcher [6].

Even though the fusion index has become a well-accepted outcome parameter to quantify the myogenic potency, more quantifiable features of myotubes and myonuclei may provide a more complete picture of the altered stem cell behavior. Indeed, earlier studies [6, 7] described additional differences between children with CP and TD children by visually comparing images from TD and CP cultures, which should be further quantified. In cell cultures, nuclei co-localize and form elongated clusters inside myotubes [12, 13]. This nuclear behavior is especially of interest, as the number of clusters, their size, and their linearity seem to differ between CP and TD children [6], and improper nuclear positioning has been linked to several muscle diseases and muscular dystrophy [13–15]. Moreover, muscular dystrophy is associated with muscle weakness [16], one of the main clinical symptoms of CP [17]. The nuclei cluster features can be described using two new parameters: number of clusters and average root mean square error (RMSE) of all clusters. Earlier studies on CP and Duchenne muscular dystrophy suggested that the number of myotubes, their shape, their size, and the number of branches originating from a single myotube were altered as well [6, 7, 18]. These features may be quantified by three other new parameters: number of myotubes, number of branching points and myotube coverage.

To facilitate and standardize the definition of all relevant parameters to quantify the myogenic potency of in vitro cell cultures, we developed an open-source MATLAB (MATLAB R2021a, MathWorks) app, the Myotube Analyzer. This allows researchers to quickly and easily determine fusion index, and the cluster- and myotube features mentioned earlier, through a semi-automatic analysis protocol. Nearly all analysis steps in the app can be done automatically, combined with the option for manual corrections. The app is open source, although editing the source code is only possible for users with a MATLAB license. Usage of the app is free and runs on MATLAB Runtime Compiler (version 9.10). The source code, the installer, the instruction manual, and analysis examples are available on GitHub [19].

This study aimed to implement the Myotube Analyzer and define the reliability and validity of the extracted outcome parameters, based on microbiopsy data of children with CP and age-related TD children. The parameters were expected to have different values for CP and TD data.

## Implementation

### Myotube Analyzer functions

Users perform the analysis using the app step-by-step. An instruction manual, a detailed definition of all outcomes and an example analysis can be found in the GitHub repository [19]. The output of the app is saved in the same folder as the input images and consists of several images saved as PNGs in different steps of the analysis, as well as an Excel file with separate tabs for each step of the analysis. All output files are named after the input images, with a suffix specifying which function produced the output. The analysis is modular, meaning that each step can be revisited without having to redo all prior steps, and that some steps can be skipped or performed at a later stage.

Before analysis, an image set consisting of JPEG or PNG images must be selected. There are three channels available: blue is used for Hoechst (nuclei), red for MyHC protein (myotubes), and, optionally, green can be used to label nuclei which are positive for a certain marker (i.e., MYOD, a myoblast transcription factor, in this case).

**Fig. 1:**
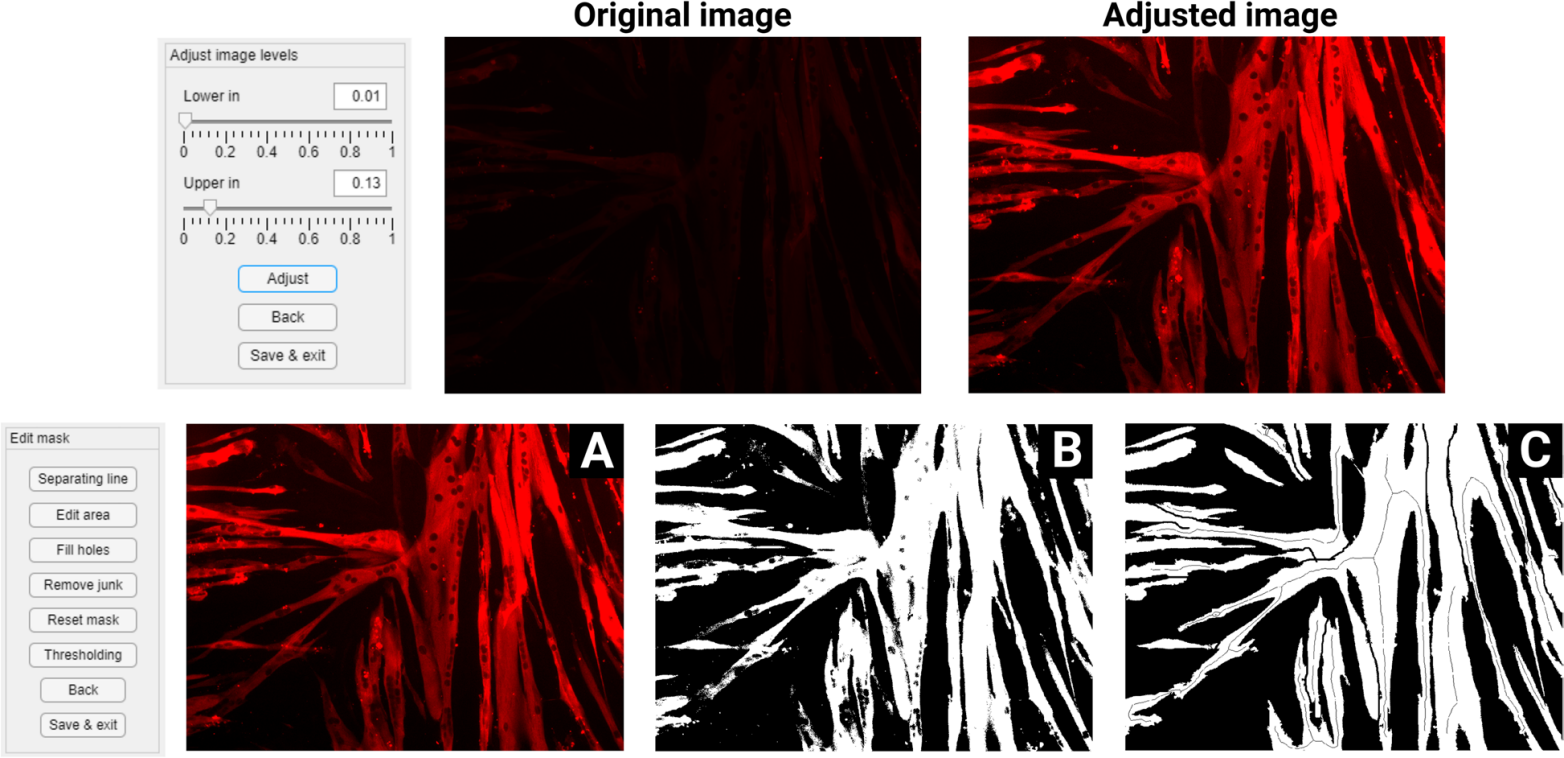
Adjusting image levels and mask editing. The upper panel shows the input, output and controls of the “Adjust levels” function. The lower panel shows the input, output and controls of the “Edit mask” function. The user first makes a rough mask (**B**) of the adjusted image (**A**) using regular thresholding. The rough mask is then edited using the editing functions to produce a mask ready for analysis (**C**)

The “Adjust levels” function makes use of an intensity windowing operation on the image histogram [20]. The histogram of each image can be adjusted to make the structures in the images visible, increase contrast, and decrease background staining (Fig. 1). This allows a reduction in exposure time and thereby avoids bleaching the cells during imaging. An input intensity range is specified by the user, and the pixels in this range are spread out over the whole possible intensity range of the image (e.g., 0–1). Adjusted images are saved as PNG files, which are used in all further steps of the analysis. Repeated use of the function will overwrite the previous adjusted image.

The “Edit mask” function has been implemented to make a binary image that indicates which parts of the red channel are myotubes and which are not. Segmenting the image is done manually using a threshold, as the pixel intensity depends on the varying expression levels of the protein and on the equipment and settings used for imaging. The resulting binary image can be edited using the various editing tools [1] and is preferential for a correct analysis. Areas can be drawn on the image to add/remove parts of myotubes, lines can be drawn to separate/join myotubes, and junk (white objects < 1000 pixels) can be removed and holes (sets of black pixels that do not touch the image border) can be filled. The mask is saved as a PNG file, where every separate myotube is indicated in a different color. This manual mask editing is crucial for indicating separate myotubes and consequently assessing all parameters using the following functions.

**Fig. 2:**
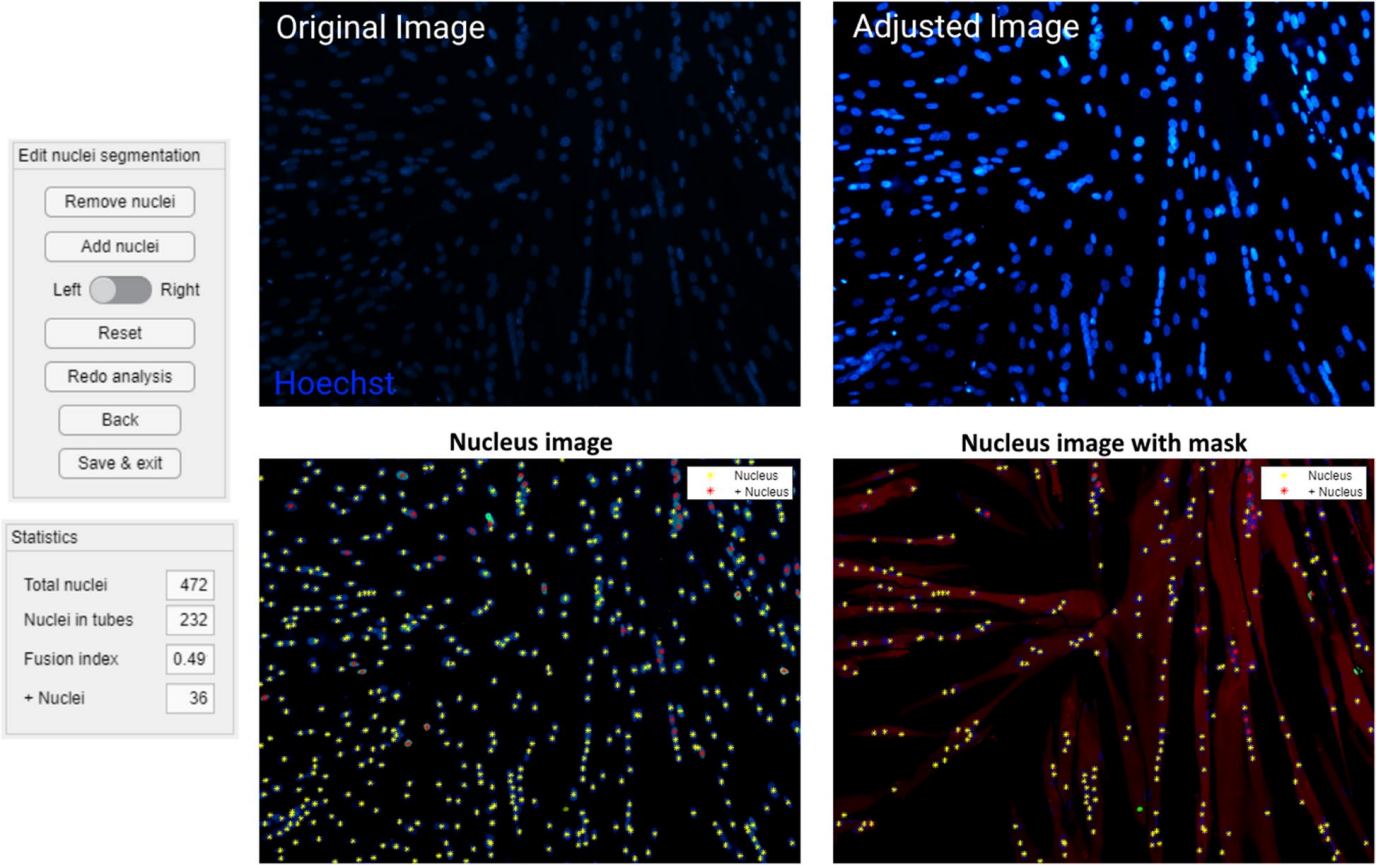
Nucleus indication. The upper panel shows both the original input image, as well as the “adjusted” image in the blue channel (nuclear staining using Hoechst). The lower panel shows editing controls, statistics panel and output of the “Nuclei indication” function. Nuclei are indicated with yellow asterisks and with red asterisks if they are positive for the marker in the green channel (due to the MyoD staining in this case). The right image does not show nuclei outside of the mask, meaning outside of the myotubes (based on MyHC, red channel). Centroids can be added or removed with the editing functions, using either the left or the right image as a guide

**Fig. 3:**
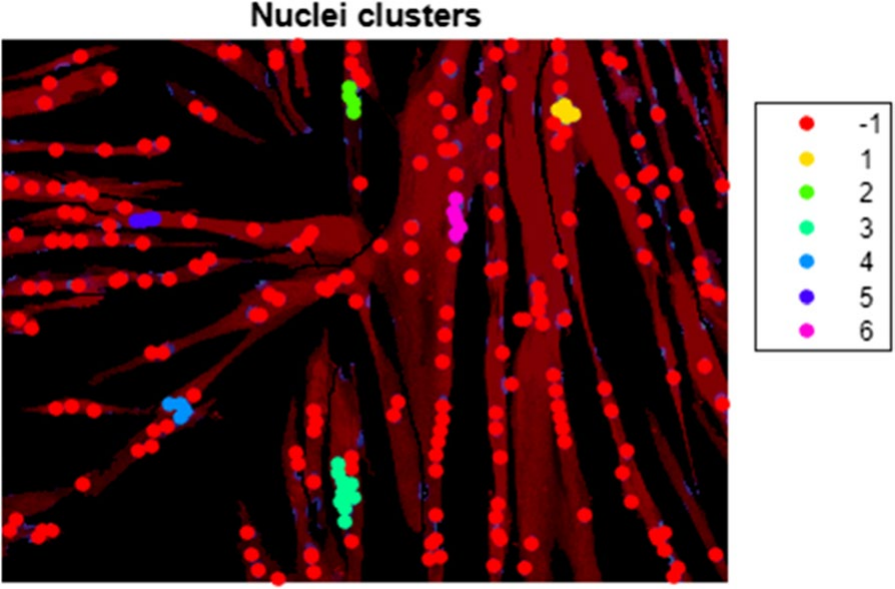
Nucleus clustering. Output of the “Cluster nuclei” function. Nuclei centroids receive a color based on their cluster assignment, with red (− 1) indicating nuclei centroids that do not belong to a particular cluster. Myosin heavy chain expression is shown in red

The “Indicate nuclei” function provides initial indications for the nuclei centers, based on the centroids of objects segmented from the blue channel (nuclear staining by Hoechst, Fig. 2), and asks the user to input which pixel size is applied in all analyses, allowing the use of images made with different microscopes and magnifications. This segmentation uses a circular filter with a radius close to that of an average nucleus as a starting point for watershed segmentation [21], which provides the objects used for the initial centroid indication. Average nucleus diameter was determined based on the distance transform [22] and regular watershed segmentation on loose nuclei in the image sets. Averaging the small and large diameter of the mostly ellipse-shaped objects obtained in this way and scaling them for the applied pixel size resulted in an average nucleus diameter of around 10 μm. Adding or removing centroids in the program is possible through the available editing functions, both on the single blue (Hoechst) channel image and the image combining the blue and red (MyHC) channel. The mask created in the previous function allows for the marking of nuclei inside MyHC-positive myotubes, so that the fusion index can be calculated and manually adjusted as previously mentioned. The green channel image (if selected) is also segmented using a fixed intensity threshold, and the program indicates the nuclei inside the resulting mask as positive for the used marker (Fig. 3). The fusion index and other statistics (total number of nuclei, number of nuclei in myotubes, total number of marked nuclei, number of marked nuclei in myotubes) are saved to an Excel output file, along with the coordinates of all individual nuclei.

The “Cluster nuclei” function aims to quantify the clustering features of the nuclei. The function uses the coordinates of the nuclei obtained in the previous function to cluster the nuclei (Fig. 3) and subsequently perform a trendline analysis on the detected clusters. The trendline is calculated using orthogonal regression, and the RMSE resulting from this calculation is used as a measure for linearity. A nucleus cluster was arbitrarily defined as a group of at least four nuclei, and clustering is performed by an agglomerative hierarchical clustering algorithm [23]. The clustering algorithm starts out by considering each nucleus as a separate cluster and looking for the two closest clusters, i.e., those that have the shortest distance between two of their elements. The algorithm then merges these clusters and repeats until the shortest distance between two clusters goes above a fixed threshold. This threshold is set by adding the nucleus diameter and the largest allowed edge-to-edge distance between nuclei. In this study, the value was set at 14 μm, allowing a maximum distance of 4 μm between the edge of a nucleus in an existing cluster and the edge of a nucleus to be added to said cluster. Edge-to-edge distance between nuclei inside a cluster can be higher, as long as one other nucleus is within this maximum distance. The maximum allowed distance, as well as the nucleus diameter, can be changed before running the clustering algorithm. The descriptive parameters of the clusters and the regression outputs are saved to a separate tab in the Excel output file. The plot of the clusters shown in 3 is saved as a PNG file and includes a color legend to visualize all clusters separately, with red indicating nuclei that do not belong to a particular cluster (labelled “ − 1”).

**Fig. 4:**
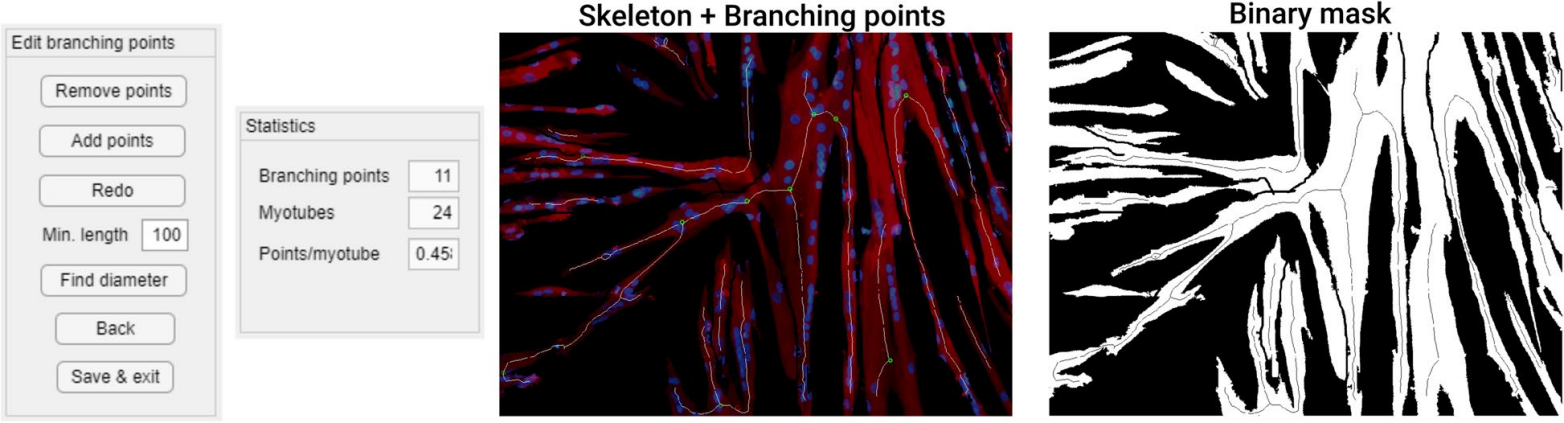
Branching points. Editing functions, statistics panel and output of the 'Branching points' function. Branching points are marked with a green circle, and can be removed or added using the editing functions. The myotube skeleton is shown in white on the left image and in black on the right image. Myosin heavy chain is shown in red, nuclear staining Hoechst in blue

**Fig. 5:**
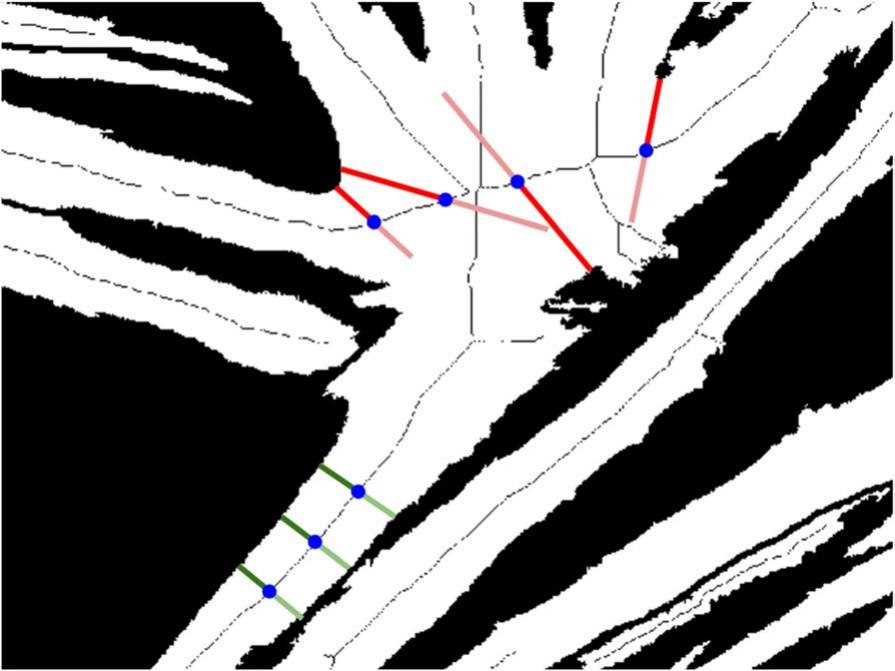
Diameter measurements. Example of diameter measurement point sampling on a myosin heavy chain mask. The calculated myotube diameter (double of the radius) at different sampling points (blue) is shown for poor sampling points (red) and good sampling points (green). The radius of the myotube as calculated by the distance transform is shown using a brighter color

**Fig. 6:**
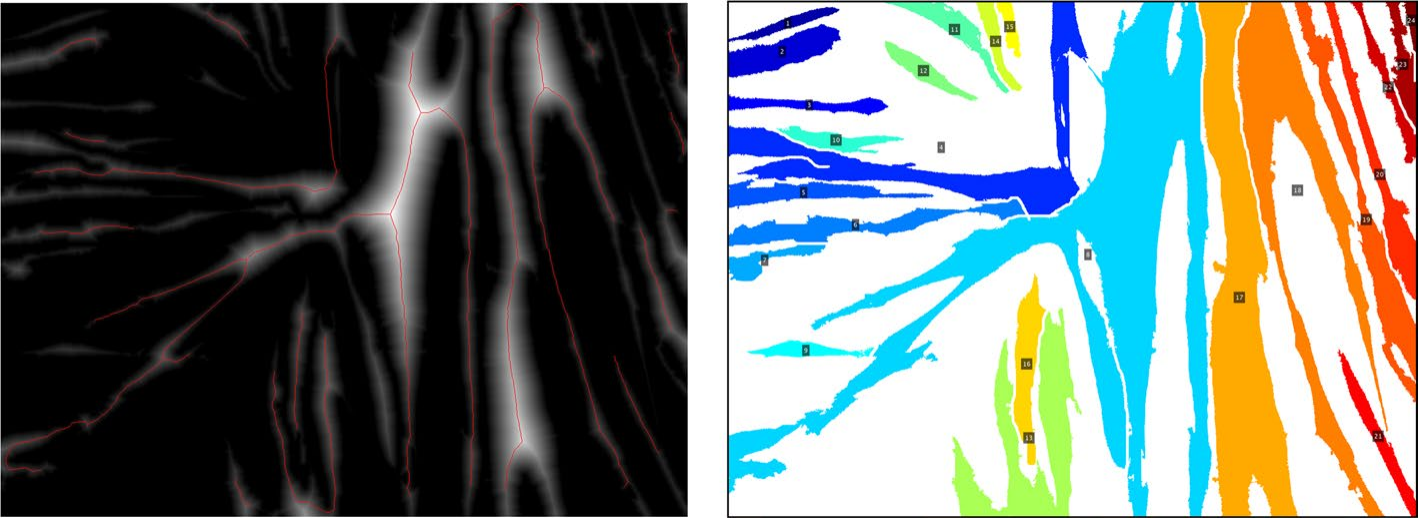
Branching points output. Image used for diameter measurements (left) and mask with labelled myotubes (right). Red lines on the left image show the myotube skeleton. Myotube labels are not always located on the myotube, since they are placed in the centroid of the myotube, which may be located outside the myotube, or even on a different myotube

The “Branching points” function provides an initial indication for the branching points in the myotubes, based on branching points in the myotube skeleton obtained using the built-in MATLAB function “bwskel” (Fig. 4). Branching points can be added or removed using the editing functions. The “Branching points” function also has the option to do diameter measurements. Points for measurement are indicated on a separate image containing the distance transform of the mask. The values of the transformed pixels contain the distance to the closest black pixel, meaning that a pixel in the middle of a myotube contains the myotube radius at that point. The user can select a set of pixels, and for each pixel, the value of the closest pixel that belongs to the myotube skeleton is doubled to obtain an estimate of the diameter. Using the pixels of the myotube skeleton gives the best possible estimate of the diameter, while also eliminating errors due to imprecise selection. Point selection is important, since the distance will no longer be measured perpendicular to the length of the myotube in the presence of myotube intersections and some myotube features, as illustrated in Fig. 5. Descriptive parameters (number of branching points, myotube coverage, number of myotubes, points per myotube), branching point coordinates, and diameter measurements are saved to separate tabs in the Excel output file. The image used for diameter measurements and a version of the mask with labels for separate myotubes are saved as separate PNG files (Fig. 6).

### Muscle microbiopsy data collection

The protocol for muscle microbiopsy collection, as well as the procedures for cell culture, immunofluorescent staining, and imaging were previously described [6]. The satellite cells were extracted from microbiopsy samples of the *Medial Gastrocnemius* muscle from five patients with CP and three age-matched TD children, all aged between 4 and 9 years (mean age TD: 5.51 ± 1.46 years, CP: 7.88 ± 0.99 years). All included patients were diagnosed with spastic bilateral cerebral palsy and Gross Motor Function Classification System levels II or III. Therefore, by keeping the same conditions previously described, this study is based on human satellite cell differentiation, seeded at 60 000 cells/cm^2^ and fixed with 4% of paraformaldehyde (Eastman Kodak) at day 6. Immunofluorescent images were obtained using an Eclipse Ti microscope (Nikon), representative for the well. Nuclei were captured in blue, using Hoechst (1:3000, Thermofisher Scientific) and myotubes in red, using an anti-myosin heavy chain antibody (MyHC, mouse, 1:20, Hybridoma Bank). All analyses performed with the app were carried out following the guidelines described in Additional file 1.

### Experimental setup

A dataset comprised of 19 image sets, each consisting of immunofluorescent staining images for nuclei (using Hoechst) and myotubes (MyHC), was used to test the feasibility of the novel app and to define the inter-rater reliability and the known-group validity for a series of outcome parameters related to nucleus and myotube properties. The inter-rater reliability was defined using intra-class correlation coefficients (ICCs) and standard errors of measurement (SEMs). Six image sets were obtained from satellite cells of TD samples and 13 from CP samples. Subdividing the dataset in this way allowed a power of > 90% for ICCs higher than ~ 0.6 when considering the whole dataset and ICCs higher than 0.7 when considering only CP samples [24]. The CP dataset was more extended, since lower ICCs were expected due to patient heterogeneity. All image sets were analyzed by two cell biologists, specialized in cell culture analysis, using the newly developed Myotube Analyzer. To define inter-rater reliability, ICCs, SEMs, and the corresponding confidence intervals were calculated using a custom MATLAB script with the formulas provided in [25–27]. The minimal detectable differences (MDDs) were calculated as $\mathrm{SEM}*1.96*\sqrt{2}$ [28]. The known-group validity was defined by comparing outcome parameters from children with CP to TD data. For each group, the median and inter-quartile range was defined. To test whether the hypothesized differences between TD and CP were quantified by the novel nucleus and myotube parameters, measurements from one analyzer were used to compare between-group differences using an unpaired two-tailed *t*-test. Statistical analyses were performed in JMP (SAS), with a significance level of 95%. In figures, the symbol “*” indicates a *p* value less than 0.05, “**” indicates *p* < 0.01, and “***” indicates *p* < 0.001. For each parameter, we also checked whether the observed significant differences exceeded the MDDs. An average difference that was larger than the MDD for a particular parameter indicated that the difference between TD and CP should be detectable in at least 95% of cases (when using an equal sample size). If not, the difference may not be large enough to distinguish from inter-rater variance, and will be detected in less than 95% of cases. An average difference that was smaller than the SEM indicates that it would be nearly indistinguishable from inter-rater variance. To comprehensively describe the features and potential added value of the semi-automatic approach of the Myotube Analyzer tool, we also explored its agreement with a fully manual approach for the parameters fusion index, number of clusters, myotubes, and nuclei. This inter-method analysis was performed on the same dataset of 19 images that was used to define the inter-rater reliability and was also based on the reliability indices ICC and SEM. For this inter-method analysis, the fully manual and the semi-automatic approach was always performed by the same rater.

### Parameter definition

Table 1 contains an overview of the definitions of each outcome parameter. All parameter calculations were implemented in the Myotube Analyzer. RMSE values and myotube coverage were also investigated for all individual clusters and myotubes, respectively.

**Table 1:** Definitions of outcome parameters

Parameter	Definition
Nucleus properties	
Average RMSE	Average RMSE was calculated from orthogonal regression after nucleus clustering, with each data point representing the average value for all the clusters in one image set. It serves as an indicator for cluster linearity
Number of clusters	The number of clusters was defined as the total number of clusters remaining after clustering
Fusion index	The fusion index was calculated for each image set as the ratio between the number of nuclei inside MyHC-positive myotubes and the total number of nuclei present in the image
Myotube properties	
Myotube coverage	Myotube coverage was calculated for each image set as the percentage of image pixels occupied by myotubes (positive pixels for MyHC in the mask) and serves as an indicator for myotube size
(Number of) branching points	The number of branching points was determined as the number of branches splitting off from one myotube, with each data point representing the sum of all branching points (for all myotubes combined) in the image set
Number of myotubes	The number of myotubes was defined as the number of separate objects in the myotube mask
Myotube diameter	Myotube diameter was defined as the average of 5 separate measurement points. Each measurement is calculated by finding the closest point on the myotube skeleton to the measurement, obtaining the intensity value from the corresponding pixel in the distance transform image, and doubling it to obtain the diameter

## Results

**Fig. 7:**
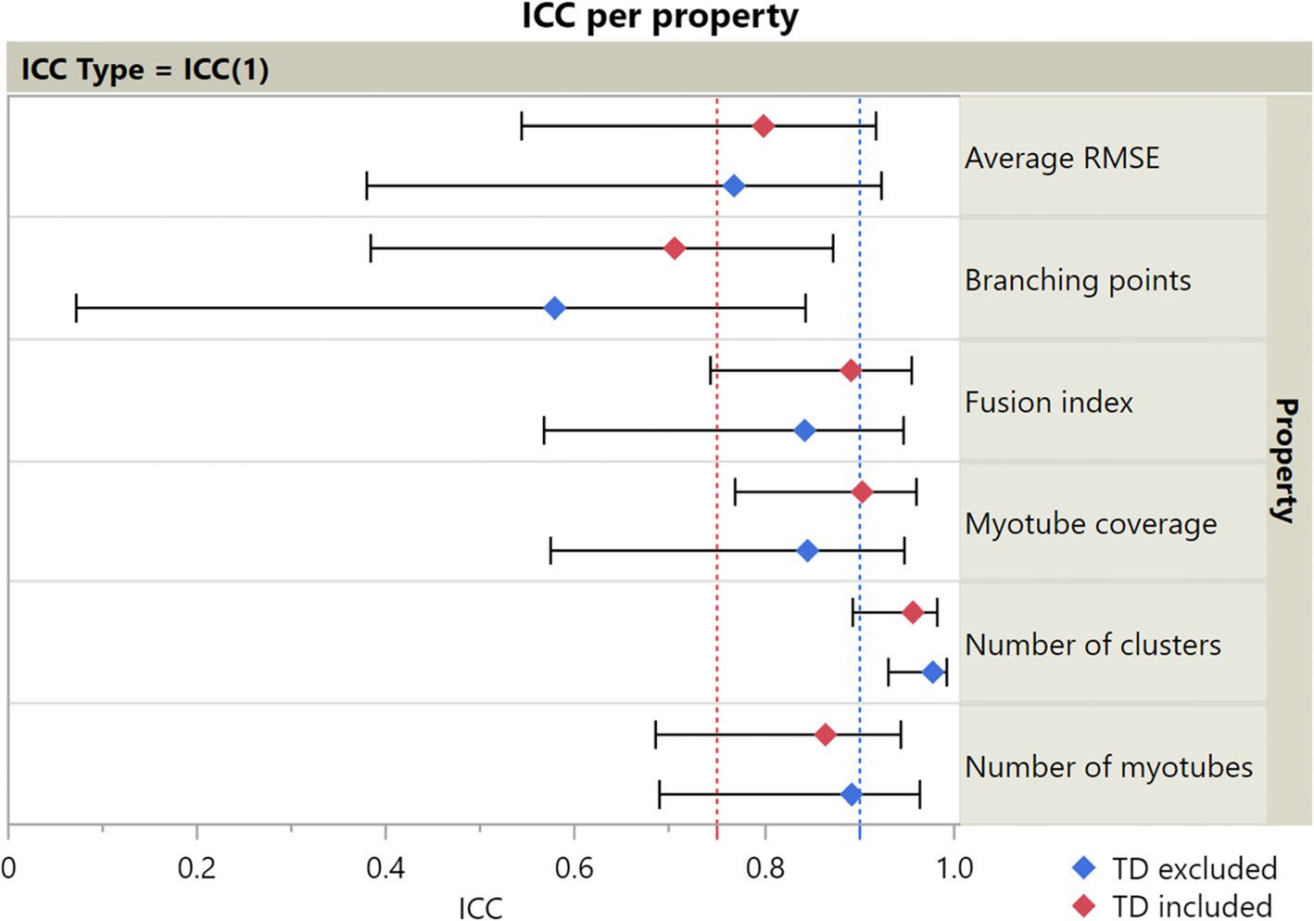
ICC values. ICC(1) calculated between two analyzers for each parameter, both including and excluding TD data points from calculation. 95% confidence intervals shown. Red line indicates values above 0.75 (good reliability); blue line indicates values above 0.9 (excellent reliability). TD, typically developing

**Fig. 8:**
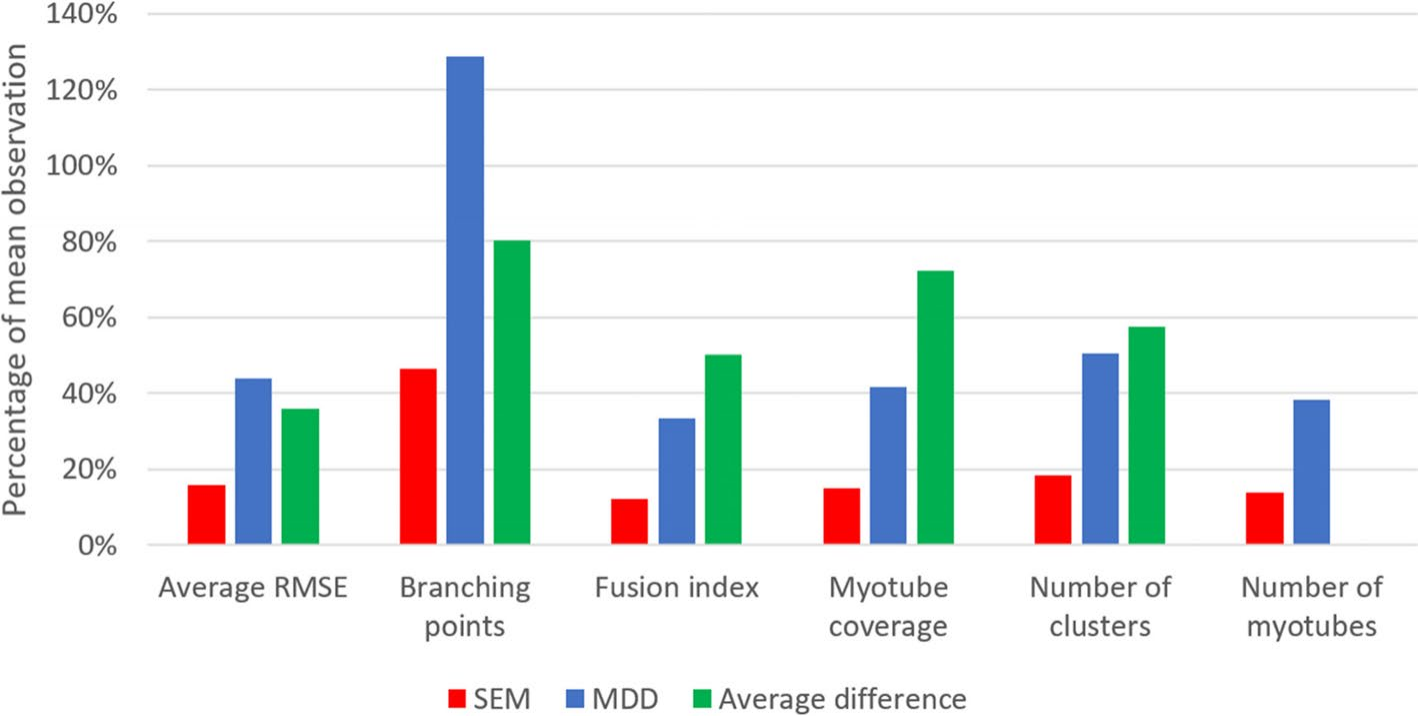
SEM, MDD, and average difference comparison. Comparison of SEM, MDD, and difference between TD and CP as percentages of the mean observation. The difference for the number of myotubes is not shown, since it was smaller than 1% of the mean observation. TD, typically developing; CP, patient with cerebral palsy

The parameter “myotube diameter” was not included in this experiment, as preliminary testing revealed that results were too subjective and variable to compare between TD and CP image sets. Figure 7 shows ICC(1) values and the corresponding 95% confidence intervals for each parameter. Values for ICC(A,1) and ICC(C,1) showed much similarity and can be found in Additional file 2. ICC values were calculated both including and excluding TD data points, since CP data were expected to show more variability. Numeric values for ICCs and SEMs are presented in Table 1. Figure 8 shows a comparison of SEMs, MDDs, and average difference between TD and CP for each parameter. Additionally, we defined the agreement between a fully manual approach and the semi-automatic approach of the Myotube Analyzer tool for the parameters fusion index, number of clusters, myotubes, and nuclei. Most ICCs were > 0.9, indicating excellent agreement between both analysis methods, while “number of clusters” had a value of > 0.75, indicating good agreement (Additional file 3). This latter parameter was slightly differently defined following a fully manual approach (based on edge-to-edge distance of maximum 4 μm between separate nuclei) versus the semi-automatic approach (based on the center-to-center distance of maximum 14 μm), which could explain the lower ICC and higher SEM values (Table 2).

**Table 2:** ICC and SEM values

Parameters	Whole dataset		TD excluded	
	ICC	SEM	ICC	SEM
Average RMSE	0.799	15.8%	0.768	12.8%
Branching points	0.705	46.5%	0.578	42.9%
Fusion index	0.892	12.0%	0.843	12.5%
Myotube coverage	0.904	15.0%	0.846	14.9%
Myotubes	0.865	13.8%	0.892	13.7%
Number of clusters	0.957	18.2%	0.978	12.5%

ICC(1) values and SEM values as a percentage of the mean observation for each parameter, calculated between two analysis methods (Myotube Analyzer and fully manual), both including and excluding TD data points from calculation

**Fig. 9:**
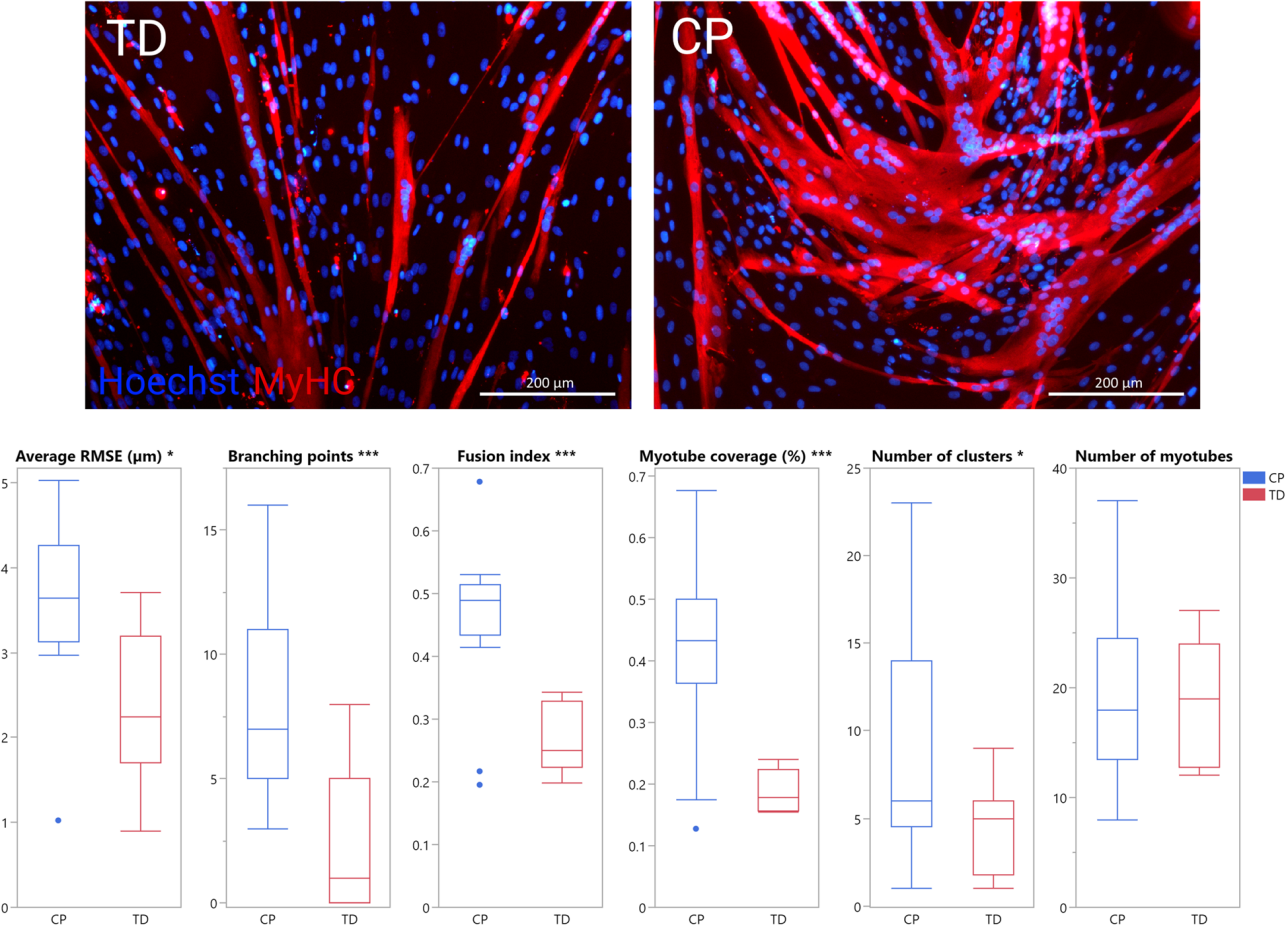
Comparison of outcome parameters for patients with CP and TD children. Representative immunofluorescent images for patients with cerebral palsy (CP) and typically developing (TD) children are shown. Myosin heavy chain (MyHC) is shown (red), nuclei are counterstained using Hoechst (blue). Scale bars are 200 μm. Boxplots of all parameters between CP (blue, *n* = 13) and TD (red, *n* = 6) image sets. Parameters where differences were significant are indicated with asterisks

Figure 9 shows a comparison between TD and CP image sets for each feature, as determined by one analyzer. Significant average differences between TD and CP are visible for all features, except for the number of myotubes (*p* < 0.05). Satellite cell-derived myotubes from patients with CP showed a higher degree of branching and larger myotube coverage. Myonuclei from CP subjects showed more clustering as well as higher average RMSE values, meaning the nucleus clusters were less linear. The fusion index was significantly higher in CP cell cultures compared to TD. For myotube coverage, the number of myotubes and the number of clusters, variance was higher for CP compared to TD image sets.

**Fig. 10:**
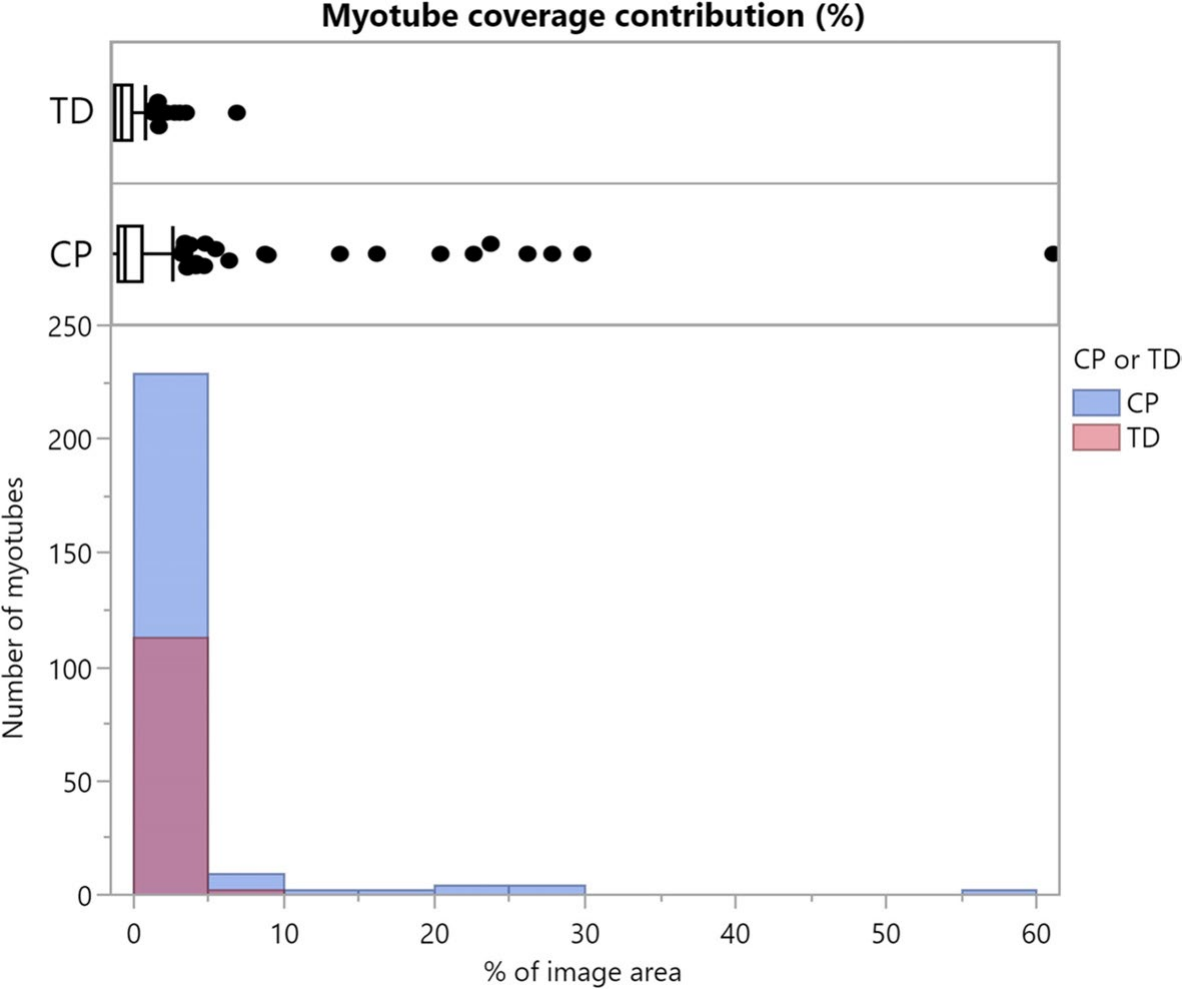
Myotube coverage contributions. Boxplot and histogram comparison between typically developing children (TD; red, *n* = 113) and patients with cerebral palsy (CP; blue, *n* = 245) of the myotube coverage contributed by each myotube (calculated per image set)

Analysis using the Myotube Analyzer app revealed a total of 139 clusters across all image sets. The RMSE of clusters found in CP image sets had a much higher variance and a higher average value (*p* < 0.001). A total of 358 myotubes were identified across all image sets. Figure 10 shows a comparison between TD and CP images for the percentage of myotube coverage contributed by each myotube (calculated per image set). Large myotubes were much more common in CP image sets, with individual myotubes from TD image sets always contributing less than 10% coverage.

## Discussion

The Myotube Analyzer allows researchers to analyze myogenic features of satellite cell cultures using not only the known and previously reported parameter fusion index but also a series of new parameters with the ability to better describe and characterize specific aspects of myotube differentiation in vitro. Myoblast cell cultures have shown to be a useful model to study multiple myopathies and for drug testing, predicting the myogenic properties for regeneration in the muscle [29–31]. Despite their broad application potential, these in vitro cell cultures have some important limitations such as the lack of stimuli from their muscle niche and other involved cell types in the complex regeneration processes [32, 33]. The tool ensures that researchers can still perform established analyses on fixed in vitro cultures, while providing the ability to perform novel analyses as well, all within a single program. All output data is conveniently grouped in one Excel file, allowing researchers to perform the data analysis with whichever statistics toolbox they prefer, while the included raw data allows for the calculation of additional parameters. The semi-automatic nature of the program ensures a quick and robust analysis, while maintaining the ability to perform the analysis entirely manually. In this light, the reliability indices (ICC and SEM values) for the agreement amongst the fully manual assessments and the proposed semi-automatic tool showed good to excellent agreement for the fusion index, number of myotubes, clusters, and nuclei. The program only requires the freely available MATLAB compiler to run, making it available for everyone, free of charge. The open-source nature of the software and the multitude of different calculated variables makes it a very flexible tool, allowing users to adapt it to their specific pathologies, species, cell types, including, i.e., mesoangioblasts and induced pluripotent stem cells, cell densities, and conditions. Moreover, analysis using the Myotube Analyzer is fairly intuitive, making it a good starting point for researchers new to this type of analysis.

As previously mentioned, the parameter “myotube diameter” was excluded from the reliability analysis because it was considered challenging to standardize the definition of the parameter and to avoid subjective interpretation. Estimating its reliability requires a protocol to determine locations for measurement point sampling. For all other parameters, with the exception of the number of branching points, ICC values were above 0.75. Some ICC values exceeded 0.9, indicating good and excellent inter-rater reliability [34]. However, the comparison of the MDDs and average differences between TD and CP data shows that a difference in average RMSE or the number of branching points may not always be detectable for this sample size. ICC(C,1) values were slightly higher than ICC(1) values for average RMSE, fusion index, and myotube coverage, indicating that these parameters were consistently higher/lower for one analyzer compared to the other. A higher value for the fusion index and myotube coverage could be explained by a mask creation threshold that is consistently set higher or lower by one analyzer. For example, a lower brightness setting on the computer display might cause a researcher to make the images brighter when adjusting them, giving a slightly different result after thresholding. These findings highlight the importance of proper training and the need for a standardized thresholding method that remains stable within experiments and that is comprehensively reported for each experiment.

With the exception of the number of myotubes, the described myotube and cluster parameters quantify the observed differences between TD and CP very well, as evidenced by the boxplots and results from the *t*-tests (Fig. 9). These findings are in line with previous qualitative observations by Corvelyn et al. [6]. However, the difference in the number of branching points and average RMSE may not always be detectable, as mentioned in the previous section (Fig. 8). Adding more image sets to increase the sample size (and therefore the power of the analysis) can mitigate this problem. Using individual cluster data may be a more suitable approach than averaging RMSE values, especially when few image sets are available. While the average number of myotubes did not significantly differ between TD and CP data, the variance appeared to be larger for the number of myotubes in CP samples. The comparison of individual myotube sizes in Fig. 10 indicates that myotube size distribution may also be different between satellite cell samples of TD subjects and patients with CP. It should be noted that all trends discussed here are based on the measurements of one analyzer, but the same trends were confirmed in the analysis of the second analyzer.

While the Myotube Analyzer is a powerful tool, it has some limitations. Due to the large number of manual inputs that can be made, the app requires some practice before analyses can be performed quickly and accurately. Manual inputs are especially necessary in the mask creation step, i.e., separating overlapping myotubes, making it the most time-consuming and subjective part of the analysis. To aid this process, and to standardize it as much as possible, an instruction manual, guidelines, and examples have been made available on GitHub. Investigating more advanced segmentation methods could potentially reduce the number of manual inputs. The use of images in TIFF-format is not supported in the app, due to an incompatibility with the MATLAB Image Processing Toolbox. However, the app does support the common PNG and JPEG formats. Since the app is open source, any shortcomings may be addressed by users within the possibilities of the MATLAB app designer.

## Conclusion

We introduced five new parameters for investigating in vitro myogenic features of satellite cells and provided a software package to measure them in a robust and reliable manner. The Myotube Analyzer app provides users with a powerful tool to determine nucleus and myotube characteristics, regardless of the pathology, species, or cell type being studied, while also serving as a framework to create new functions or to modify existing ones. The results of the known-group validity analysis confirm that most of the hypothesized differences in these features between TD and CP data can be quantified using the proposed parameters. Semi-automatic analysis with the Myotube Analyzer app by two analyzers was found to have little inter-rater variability for all parameters, except for the number of branching points. Evaluation of SEM and MDD values showed that three out of six studied parameters based on in vitro satellite cell differentiation could be used to reliably show differences between TD and CP image sets.

## Availability and requirements

Project name: The Myotube Analyzer: how to assess myogenic potency in human adult muscle stem cells.

Project home page: https://github.com/SimonNoe/myotube-analyzer-app

Operating system(s): The source code is platform independent, though the standalone (MA_Installer.exe) will only work on Windows machines.

Programming language: MATLAB.

Other requirements: None.

License: CC BY-NC 4.0

Any restrictions to use by non-academics: Only non-commercial use allowed.

## Supplementary Information

**Additional file 1.** Guidelines for analysis with the Myotube Analyzer. These guidelines were decided on after a few pilot experiments on different image sets, and iterations of multidisciplinary discussions involving the program developer and the cell culture specialists.

**Additional file 2.** Figure of all ICC and SEM values. ICC(1) is a general indicator of reliability/consistency. ICC(A,1) is an indicator of absolute agreement, meaning that small differences (in absolute value) between analysers results in a high value. ICC(C,1) is an indicator of relative agreement, meaning that little variation in the differences between analysers results in a high value. If ICC(C,1) is higher than the other two values, some form of bias might be present in the measurements. SEM is an estimate for standard error.

**Additional file 3.** Figure ofg ICC and SEM values comparing the Myotube Analyzer and fully manual analysis. The upper panel shows the visualization of ICC(1) calculated between two analysis methods (Myotube Analyzer and fully manual) for multiple parameters, both including and excluding TD data points from calculation. 95% confidence intervals are shown. The red line indicates values above 0.75 (good reliability), the blue line indicates values above 0.9 (excellent reliability). The lower panel shows the table of the ICC values and SEM values as a percentage of the mean observation for each parameter. TD: typically developing, CP: cerebral palsy (CP: *n* = 13, TD: *n* = 6)

## Data Availability

The datasets used and/or analyzed during the current study are available from the corresponding author on reasonable request.

## Abbreviations

CP: Cerebral palsy
ICC: Intra-class correlation coefficient
JPEG: Joint photographic experts group image format
MDD: Minimal detectable difference
MyHC: Myosin heavy chain protein
PNG: Portable network graphics image format
RMSE: Root mean square error
SEM: Standard error of measurement
TD: Typically developing

## References

[1] Rosenbaum Peter, Paneth Nigel, Leviton Alan, Goldstein Murray, Bax Martin (2005). Proposed definition and classification of cerebral palsy. Dev Med Child Neurol..

[2] Barrett RS, Lichtwark GA (2010). Gross muscle morphology and structure in spastic cerebral palsy: a systematic review. Dev Med Child Neurol..

[3] Mathewson MA, Lieber RL (2015). Pathophysiology of Muscle Contractures in Cerebral Palsy. Phys Med Rehabil Clin North Am..

[4] Dayanidhi S, Dykstra PB, Lyubasyuk V, McKay BR, Chambers HG, Lieber RL (2015). Reduced satellite cell number in situ in muscular contractures from children with cerebral palsy. J Orthop Res.

[5] von Walden F, Gantelius S, Liu C, Borgström H, Björk L, Gremark O (2018). Muscle contractures in patients with cerebral palsy and acquired brain injury are associated with extracellular matrix expansion, pro-inflammatory gene expression, and reduced rRNA synthesis. Muscle Nerve.

[6] Corvelyn M, de Beukelaer N, Duelen R, Deschrevel J, van Campenhout A, Prinsen S, et al. Muscle microbiopsy to delineate stem cell involvement in young patients: a novel approach for children with cerebral palsy. Front Physiol. 2020;11. 10.3389/fphys.2020.00945 PMC7424076 32848872

[7] Domenighetti AA, Mathewson MA, Pichika R, Sibley LA, Zhao L, Chambers HG (2018). Loss of myogenic potential and fusion capacity of muscle stem cells isolated from contractured muscle in children with cerebral palsy. Am J Physiol Cell Physiol..

[8] Catteau M, Gouzi F, Blervaque L, Passerieux E, Blaquière M, Ayoub B (2020). Effects of a human microenvironment on the differentiation of human myoblasts. Biochem Biophys Res Commun.

[9] Carvajal Monroy PL, Grefte S, Kuijpers-Jagtman AM, von den Hoff JW, Wagener FADTG (2017). Neonatal satellite cells form small myotubes in vitro. Journal of Dental Research..

[10] Nishiyama T, Kii I, Kudo A. Inactivation of Rho/ROCK signaling is crucial for the nuclear accumulation of FKHR and myoblast fusion. J Biol Chem. 2004;279(45):47311–9. 10.1074/jbc.M403546200 15322110

[11] Schindelin J, Arganda-Carreras I, Frise E, Kaynig V, Longair M, Pietzsch T (2012). Fiji: an open-source platform for biological-image analysis. Nat Methods.

[12] Gache V, Gomes ER, Cadot B (2017). Microtubule motors involved in nuclear movement during skeletal muscle differentiation. Mol Biol Cell.

[13] Cadot B, Gache V, Gomes ER (2015). Moving and positioning the nucleus in skeletal muscle – one step at a time. Nucleus.

[14] Liu J, Huang Z-P, Nie M, Wang G, Silva WJ, Yang Q (2020). Regulation of myonuclear positioning and muscle function by the skeletal muscle-specific CIP protein. Proc Natl Acad Sci.

[15] Wang Z, Cui J, Wong WM, Li X, Xue W, Lin R (2013). Kif5b controls the localization of myofibril components for their assembly and linkage to the myotendinous junctions. Development.

[16] Barro M, Carnac G, Flavier S, Mercier J, Vassetzky Y, Laoudj-Chenivesse D. Myoblasts from affected and non-affected FSHD muscles exhibit morphological differentiation defects. J Cell Mol Med. 2010;14(1–2):275–89. 10.1111/j.1582-4934.2008.00368.x PMC2910739 18505476

[17] Graham HK, Rosenbaum P, Paneth N, Dan B, Lin JP, Damiano DiL, et al. Cerebral palsy. Nat Rev Dis Prim. 2016;2:15082. 10.1038/nrdp.2015.82 PMC9619297 27188686

[18] Chal J, Oginuma M, Al Tanoury Z, Gobert B, Sumara O, Hick A (2015). Differentiation of pluripotent stem cells to muscle fiber to model Duchenne muscular dystrophy. Nature Biotechnology..

[19] Noë S. The Myotube Analyzer. https://github.com/SimonNoe/myotube-analyzer-app. 2022.

[20] Pratt WK (2001). Digital Image Processing.

[21] Beucher S, Meyer F. The morphological approach to segmentation: the watershed transformation. In: Mathematical morphology in image processing. CRC Press; 1993.

[22] Breu H, Gil J, Kirkpatrick D, Werman M (1995). Linear time Euclidean distance transform algorithms. IEEE Trans Pattern Anal Mach Intell.

[23] Bouguettaya A, Yu Q, Liu X, Zhou X, Song A (2015). Efficient agglomerative hierarchical clustering. Expert Syst Appl.

[24] Bujang MA, Baharum N. A simplified guide to determination of sample size requirements for estimating the value of intraclass correlation coefficient: a review. Arch Orofacial Sci J School Dent Sci USM Arch Orofac Sci. 2017;12:1–11.

[25] Shrout PE, Fleiss JL. Intraclass correlations: uses in assessing rater reliability. Psychol Bull. 1979;86(2):420–8. 10.1037//0033-2909.86.2.420 18839484

[26] Liljequist D, Elfving B, Roaldsen KS. Intraclass correlation – a discussion and demonstration of basic features. PLoS ONE. 2019;14(7). 10.1371/journal.pone.0219854 PMC6645485 31329615

[27] Stratford PW, Goldsmith CH (1997). Use of the standard error as a reliability index of interest: an applied example using elbow flexor strength data. Phys Ther.

[28] de Vet HC, Terwee CB, Ostelo RW, Beckerman H, Knol DL, Bouter LM (2006). Minimal changes in health status questionnaires: distinction between minimally detectable change and minimally important change. Health Qual Life Outcomes.

[29] Park S-Y, Yun Y, Lim J-S, Kim M-J, Kim S-Y, Kim J-E (2016). Stabilin-2 modulates the efficiency of myoblast fusion during myogenic differentiation and muscle regeneration. Nat Commun.

[30] Yoon JH, Lee S-M, Lee Y, Kim MJ, Yang JW, Choi JY (2022). Alverine citrate promotes myogenic differentiation and ameliorates muscle atrophy. Biochem Biophys Res Commun.

[31] Zhang H, Wen J, Bigot A, Chen J, Shang R, Mouly V, et al. Human myotube formation is determined by MyoD–Myomixer/Myomaker axis. Sci Adv. 2020;6(51). 10.1126/sciadv.abc4062 PMC11206528 33355126

[32] Murach KA, White SH, Wen Y, Ho A, Dupont-Versteegden EE, McCarthy JJ (2017). Differential requirement for satellite cells during overload-induced muscle hypertrophy in growing versus mature mice. Skeletal Muscle.

[33] Snijders T, Nederveen JP, McKay BR, Joanisse S, Verdijk LB, van Loon LJC, et al. Satellite cells in human skeletal muscle plasticity. Front Physiol. 2015;6. 10.3389/fphys.2015.00283 PMC4617172 26557092

[34] Koo TK, Li MY (2016). A guideline of selecting and reporting intraclass correlation coefficients for reliability research. J Chiropr Med.

