# Supplementary material for: The Myotube Analyzer: how to assess myogenic features in muscle stem cells
Source: Skelet Muscle. 2022 Jun 10;12:12. doi: 10.1186/s13395-022-00297-6 (PMC9185954; doi:10.1186/s13395-022-00297-6)
Supplement: Supplementary file 1 — Additional file 1. Guidelines for analysis with the Myotube Analyzer. These guidelines were decided on after a few pilot experiments on different image sets, and iterations of multidisciplinary discussions involving the program developer and the cell culture specialists. [file 13395_2022_297_MOESM1_ESM.docx]

# Guidelines for analysis with the Myotube Analyzer

These guidelines were decided on after a few pilot experiments on different image sets, and iterations of multidisciplinary discussions involving the program developer and the cell culture specialists.

**Adjust levels**

- Always choose a lower input threshold higher than 0 for DAPI to avoid that the software recognizes background signal as nuclei
- Do not oversaturate the images (else you cannot see whether 2 myotubes are overlapping, or branches of the same myotube for example)

**Indicate mask**

Thresholding: set threshold as low as possible, until noise appears

**When separating myotubes**

- A shadow or line over the whole length of the possible cut-off must be visible
- Keep the main/biggest myotube intact, don’t cut completely through the main myotube, but alongside it where the other myotube touches the main one
- When in doubt: separate the myotubes

**When adding areas**

- Dim myotubes not entirely in the mask need to have at least 2 nuclei in MyHC area **AND** 50% should be already visible in mask (if mask didn’t find them, don’t include them)

Note: Everything that the mask already clearly included, even though there might be just one nucleus, is kept

- Fill holes of nuclei in the MyHC mask
- Remove junk before saving

**Indicate nuclei**

When doubt whether or not an object is a nucleus (very big or very small, very high or very low intensity, irregular in shape): remove it

For poorly assigned nuclei (asterisk is not centered, asterisks are missing, too many asterisks)

- Less than 4 nuclei in the neighborhood: leave as is, only add missing nuclei or remove excess
- More than 4 nuclei in the neighborhood: remove and reassign centers
- When in doubt: remove and redo

**Nuclei clustering**

- Nucleus diameter: 10 µm
- Max allowed distance: 4 µm

**Branching points**

- Keep the general overview (do not zoom in)
- Do not consider bulges as branching points, there really has to be a myotube (containing at least one nucleus) in another direction
- Curves are not considered for branching points
- In case of multiple branches arriving at a point, follow the skeleton to decide the number of branching points in that area
- If visually you cannot distinguish branching points, just consider one

When in doubt: do not add a branching point)
