# Supplementary figures and images for: The Myotube Analyzer: how to assess myogenic features in muscle stem cells
Source: Skelet Muscle. 2022 Jun 10;12:12. doi: 10.1186/s13395-022-00297-6 (PMC9185954; doi:10.1186/s13395-022-00297-6)

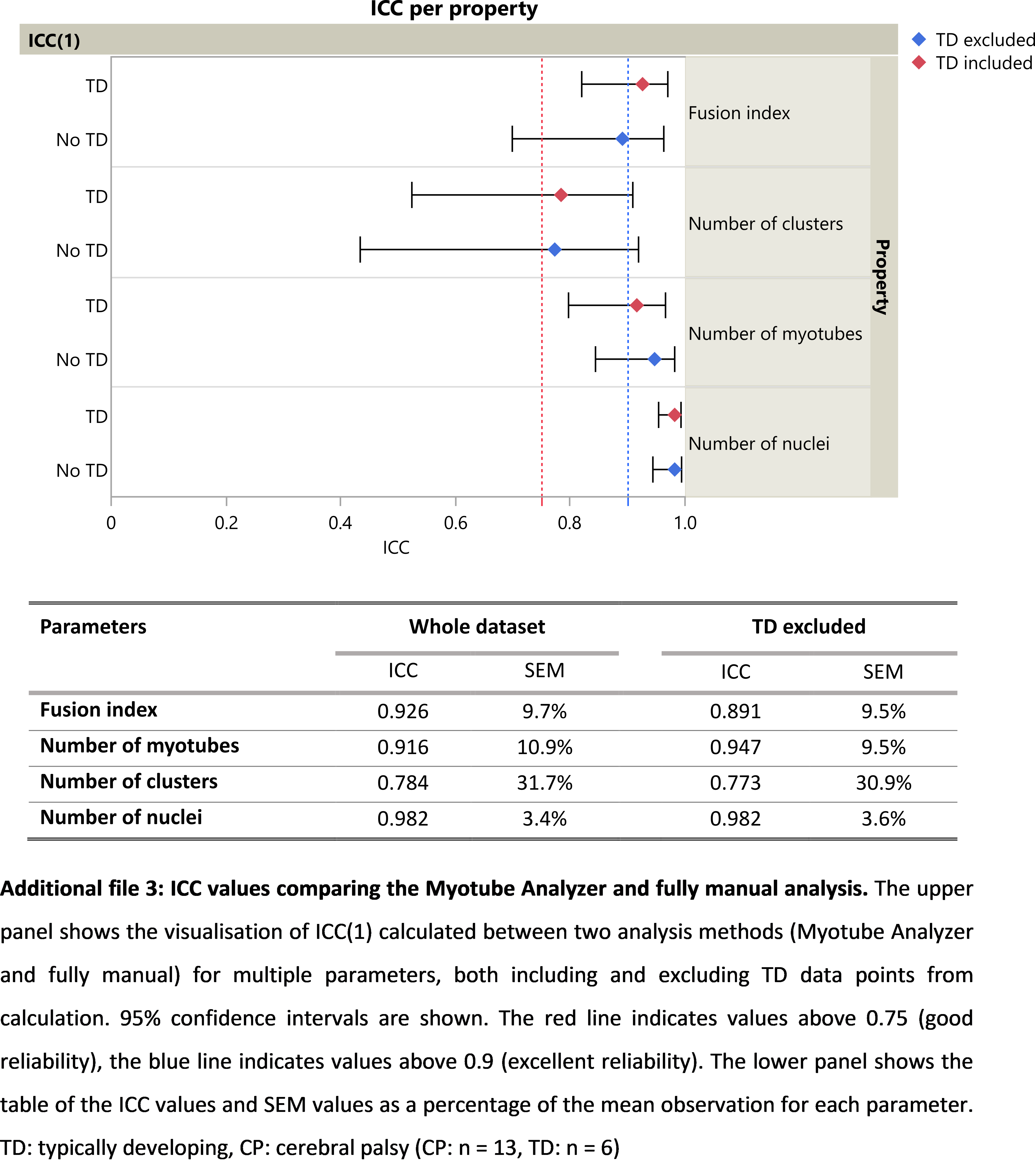

Supplement: Supplementary file 3 — Additional file 3. Figure ofg ICC and SEM values comparing the Myotube Analyzer and fully manual analysis. The upper panel shows the visualization of ICC(1) calculated between two analysis methods (Myotube Analyzer and fully manual) for multiple parameters, both including and excluding TD data points from calculation. 95% confidence intervals are shown. The red line indicates values above 0.75 (good reliability), the blue line indicates values above 0.9 (excellent reliability). The lower panel shows the table of the ICC values and SEM values as a percentage of the mean observation for each parameter. TD: typically developing, CP: cerebral palsy (CP: n = 13, TD: n = 6) [file 13395_2022_297_MOESM3_ESM.png]
